# A randomized trial of mail vs. telephone invitation to a community-based cardiovascular health awareness program for older family practice patients [ISRCTN61739603]

**DOI:** 10.1186/1471-2296-6-35

**Published:** 2005-08-19

**Authors:** Tina Karwalajtys, Janusz Kaczorowski, Larry W Chambers, Cheryl Levitt, Lisa Dolovich, Bea McDonough, Christopher Patterson, James E Williams

**Affiliations:** 1 Department of Family Medicine, McMaster University, Hamilton, Canada; 2 Department of Clinical Epidemiology and Biostatistics, McMaster University, Hamilton, Canada; 3 Social and Public Health Services Department, City of Hamilton, Canada; 4 Élisabeth Bruyère Research Institute, a University of Ottawa and SCO Health Service Partnership, Ottawa, Canada; 5 Centre for Evaluation of Medicines, Father Sean O'Sullivan Research Centre, St. Joseph's Healthcare Hamilton, and McMaster University, Faculty of Health Sciences, Hamilton, Canada; 6 Division of Geriatric Medicine, McMaster University, Hamilton, Canada

## Abstract

**Background:**

Family physicians can play an important role in encouraging patients to participate in community-based health promotion initiatives designed to supplement and enhance their in-office care. Our objectives were to determine effective approaches to invite older family practice patients to attend cardiovascular health awareness sessions in community pharmacies, and to assess the feasibility and acceptability of a program incorporating invitation by physicians and feedback to physicians.

**Methods:**

We conducted a prospective randomized trial with 1 family physician practice and 5 community pharmacies in Dundas, Ontario. Regular patients 65 years or older (n = 235) were randomly allocated to invitation by mail or telephone to attend pharmacy cardiovascular health awareness sessions led by volunteer peer health educators. A health record review captured blood pressure status, monitoring and control. At the sessions, volunteers helped patients to measure blood pressure using in-store machines and a validated portable device (BPM-100), and recorded blood pressure readings and self-reported cardiovascular risk factors. We compared attendance rates in the mail and telephone invitation groups and explored factors potentially associated with attendance.

**Results:**

The 119 patients invited by mail and 116 patients contacted by telephone had a mean age of 75.7 (SD, 6.4) years and 46.8% were male. Overall, 58.3% (137/235) of invitees attended a pharmacy cardiovascular health awareness session. Patients invited by telephone were more likely to attend than those invited by mail (72.3% vs. 44.0%, OR 3.3; 95%CI 1.9–5.7; *p *< 0.001).

**Conclusion:**

While the attendance in response to a telephone invitation was higher, response to a single letter was substantial. Attendance rates indicated considerable interest in community-based cardiovascular health promotion activities. A large-scale trial of a pharmacy cardiovascular health awareness program for older primary care patients is feasible.

## Background

Hypertension is a major contributor to cardiovascular disease and associated morbidity and mortality among older adults in Canada. The prevalence of hypertension increases with age and more than half of men and women 65 to 74 years of age have a systolic or diastolic blood pressure above the target level of 140/90 mmHg (or 130/80 mmHg if diabetes or target organ damage is present) recommended by the Canadian guidelines [1,2]. High blood pressure is an important and modifiable risk factor for heart disease, stroke, renal failure, peripheral vascular disease [3], and Alzheimer's disease [4]. Since elevated blood pressure is generally asymptomatic, it is important to find effective strategies to facilitate diagnosis and control of hypertension and promotion of the cardiovascular health in communities across Canada.

The diagnosis of hypertension is primarily made in the offices of family physicians [3]. Although provincial survey data indicate that nearly all older adults in Ontario visit their physician annually [5], there is evidence that one half to one third of patients have elevated blood pressures for which no diagnosis is recorded or treatment prescribed [6].

Effective diagnosis and control of hypertension depends on physicians' awareness of a patient's blood pressure status and cardiovascular risk profile over time [7]. Diagnosing hypertension can be complicated and frequently requires multiple assessments over time [8]. In-office monitoring may be limited by infrequent patient visits, co-morbid conditions, time or space constraints, remuneration and the presence of 'white coat', or 'masked' hypertension.

Since multiple, accurate blood pressure readings are needed to diagnose hypertension and regular monitoring is necessary for effective, on-going control of high blood pressure, accurate supplementary readings, a cardiovascular health profile, and more active involvement of patients in their care may help physicians to better manage hypertension in their practices. Blood pressure monitoring in a familiar, convenient community setting such as community pharmacies, with support from trained peer volunteers and delivery of patient-specific blood pressure readings and cardiovascular risk factor information to family physicians, pharmacists and the patients, could help overcome barriers to effective management [9].

Family physicians can play an important role in encouraging patients to participate in health promotion initiatives designed to supplement and enhance their in-office care. In a study by McAuley *et al*, a letter of invitation from the family physician was found to be an effective strategy to recruit women for routine mammography, with nearly 70% success [10]. Given that public opinion polls demonstrate that physicians are among the most trusted professionals [11], patient acceptance of new approaches to routine care may be largely dependent on their physician's endorsement and involvement. Older patients with a long relationship with their family physician may be particularly receptive to the physician's advice. A recent study recruiting older adults in family practice achieved a 70% success rate and found that the median time of patient association with the current physician was 10.2 years in the intervention group and 11.5 years in the control group, with a standard deviation of 9 years [12].

The program described here seeks to: 1) maximize use of existing resources such as community pharmacies and volunteers in cardiovascular health promotion; 2) ensure that community-based cardiovascular risk assessment is linked to family physicians who can provide follow up. The program is unique because it incorporates collection of patient-reported risk factors in addition to blood pressure readings, and 'closes the loop' by delivering results to the physician. However, there are many community programs offering blood pressure monitoring, and the idea of inviting older adults via family practices to attend a new program for cardiovascular health assessment prompted some scepticism – would they come? A first step was to determine the effectiveness of different approaches for inviting older adults to participate in pharmacy cardiovascular health awareness sessions, and the appeal of a program incorporating invitation by physicians and feedback of patient-specific results to physicians.

We conducted a randomized trial to determine the best method (mail or telephone) for family physicians' offices to invite patients to attend a session, and to identify factors that predicted attendance. In preparation for a larger scale randomized controlled trial, we also performed a cost analysis of invitation method and success rate, investigated the operational and methodological aspects of the proposed intervention, and queried patients' willingness to continue to attend community pharmacy blood pressure sessions and their preferences for the time and frequency of the sessions.

## Methods

**Figure 1:**
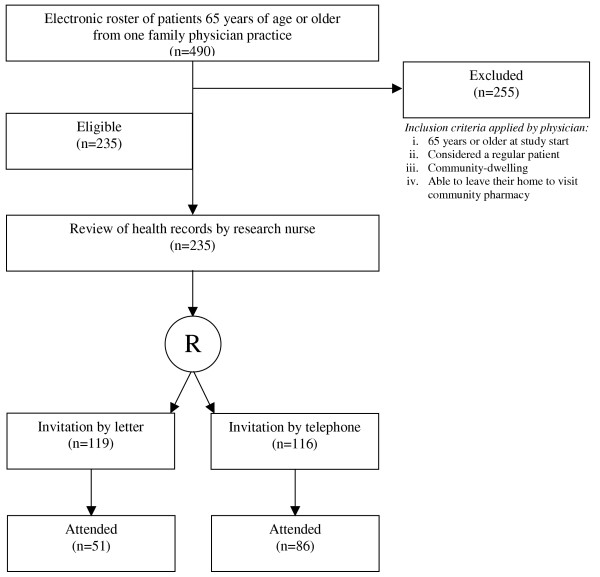
Study design and patient flow.

The study design was a prospective randomized trial (see Figure 1). The primary end-point was the overall attendance among patients invited by mail compared to those invited by telephone. Potential predictors of attendance, including cardiovascular risk factors recorded in patient health records, were assessed. The study was approved by the Hamilton Health Sciences / McMaster University Faculty of Health Sciences Research Ethics Board.

Eligible patients were identified from the practice roster of one family physician. Patients were eligible to participate if they were 65 years of age or older, considered by the physician to be regular patients, community dwelling, and able to attend a cardiovascular health promotion session in a local pharmacy. Patients were excluded if they suffered from dementia or a serious, non-cardiovascular disease or condition, or were non-English speaking and could not attend with an English-speaking companion. An electronic list of patients 65 years or older (n = 490) was generated using billing data. Of these, 235 met the eligibility criteria and were included in the trial. The list was reviewed and eligibility verified by the physician. The targeting of regular patients reflects the increasing move to rostered practices in Ontario and the potential importance of an established relationship with the provider, where mail/telephone contact related to health promotion would not be unusual.

Computer-generated random numbers were used to allocate eligible patients to invitation by mail (n = 119) or by telephone (n = 116) to attend one of five blood pressure monitoring sessions scheduled in five local pharmacies. To prevent potential contamination of the study arms, patients sharing the same address and/or surname were assigned identical identification numbers and thus randomly allocated to the same group. Mailed letters used an electronic signature from the physician. Patients assigned to the telephone group received a telephone call from a member of the physician's office staff who followed a protocol that limited repeat calls to three and included a structured script. Patients who were unreachable by either contact method (letters returned to sender or calls busy/unanswered) were included in the intention-to-treat analysis of attendance by invitation method in order to establish the feasibility of the program in real practice.

Research nurses performed a baseline health record review to confirm patient eligibility, collect demographic and health status factors that might influence session attendance, and document blood pressure monitoring and control. Data collected included age, gender, formal diagnosis or suspicion of hypertension, related physician comments, cardiovascular risk factors (diabetes, high cholesterol, smoking, family history), and all blood pressure readings recorded in the chart in the previous six months.

The five pharmacy blood pressure sessions were held during a 10-day period in April of 2001. Attending patients were asked to provide signed informed consent to participate. Patients were able to 'opt-out' of the feedback to the physician, however no participants in this small trial objected to having their results forwarded to the physician. At the sessions, volunteer Peer Health Educators trained by a public health nurse helped participants to measure their blood pressure using both a portable, automated device (BPM-100, *VSM MedTech Ltd., Coquitlam, Canada*) [13] and the in-store device, and recorded results and additional patient-reported cardiovascular risk factors. The portable device had been validated and met international standards for accuracy [13,14].

The session recording form captured, in triplicate, blood pressure readings and a checklist of 14 cardiovascular risk factors for distribution to the patient, family physician and regular pharmacist. A patient questionnaire collected demographic and general health information, current blood pressure status and history, history of related health problems, and preferences for place, time and frequency of blood pressure monitoring.

### Statistical analysis

To achieve a power of 80% to detect a difference between groups of 20% or more, the significance level (alpha) was set at 0.05 and at least 103 patients were required for each study arm. The comparability of groups was established maintaining the denominator of all patients randomized (n = 235), regardless of whether or not telephone or mail contact was successful. The probability of a Type I error (alpha) was chosen to be 0.05 (two-tailed) in all analyses.

Patients allocated to the mail or telephone contact group were compared on data collected in the health record review: median age, gender, mean blood pressure, level of blood pressure, previous diagnosis of hypertension, smoking status, family history of cardiovascular disease, high cholesterol, and diabetes. The mean of the blood pressure readings recorded in the last six months, collected in the health record review, were categorized in three levels, based on whether the systolic or diastolic pressure exceeded upper boundaries.

To determine whether attending patients systematically responded differently to the invitation method, attenders and non-attenders were compared on median age, gender, mean blood pressure, and previous diagnosis of hypertension using t-tests or chi-square tests as appropriate. We examined the potential association of factors captured in the health record review (formal diagnosis or suspicion of hypertension, related physician comments, cardiovascular risk factors, blood pressure control) with attendance at the cardiovascular health promotion sessions, using chi-square tests.

To determine what combination of variables best predicted the likelihood of patient attendance at community-based cardiovascular health promotion sessions, logistic regression was performed, controlling for allocation group. Variables entered into the stepwise model included contact method, age (65–74 yr or 75+), gender, diagnosis of hypertension, and the four cardiovascular risk factors (diabetes, high cholesterol, smoking, family history) collected in the health record review.

A retrospective cost-analysis compared the cost per one patient attending in each invitation arm.

## Results

Patients allocated to each of the experimental groups (mail and telephone invitation) were found to be comparable on median age, gender, mean blood pressure, level of blood pressure, previous diagnosis of hypertension, and four additional cardiovascular risk factors (see Table 1). Patients who attended a session and those who did not were also found to be comparable on median age, gender, previous diagnosis of hypertension, and four additional cardiovascular risk factors (see Table 2).

**Table 1:** Characteristics of 235 patients randomly allocated to mail or telephone invitation to attend a cardiovascular health promotion session.

**Patient Characteristics**		**Mail invitation n = 116(%)**	**Telephone invitation n = 119(%)**
Median Age (min 65 – max 96)	***Male***	76	77
	***Female***	76	75
			
Gender	***Male***	55 (47.4)	55 (46.2)
			
Mean blood pressure in health record (mm Hg)*		136 / 71	136 / 72
			
Formal diagnosis of hypertension*		50 (44.2)	56 (50.0)
			
Health record blood pressure readings (mm Hg)*	***≤ 140 and ≤90***	62 (54.4)	65 (57.5)
			
	***≥140 or ≥90 to ≤160 and ≤100***	38 (33.3)	36 (31.9)
			
	***≥160 or ≥100 or no readings***	14 (12.3)	12 (10.6)
			
Cardiovascular risk factors*	***Smoking***	47 (41.2)	37 (32.7)
	***Family history***	33 (29.2)	36 (31.9)
	***High cholesterol***	40 (35.4)	45 (39.8)
	***Diabetes***	17 (15.0)	22 (19.5)

* During the health record review, 8 patients were discovered to be ineligible and 1 audit was incomplete. Therefore, the denominator for health record review data ranges from 112 to 114.

**Table 2:** Characteristics of the 98 non-attending patients compared to 137 patients who attended a cardiovascular health promotion session.

**Patient Characteristics**		**Non-attenders n = 98(%)**	**Attenders n = 137(%)**
Median Age (yr; 65–96)	***Male***	76	75
	***Female***	76	77
			
Gender	***Male***	45 (45.9)	65 (47.4)
			
Mean blood pressure in health record (mm Hg)		135 / 71	137 / 72
			
Formal diagnosis of hypertension*		46 (51.7)	60 / 136 (44.1)
			
Family physician-reported cardiovascular risk factors*	***Smoking***	34 (38.2)	50 (36.5)
			
	***Family history†***	20 (22.5)	49 (35.8)
			
	***High cholesterol***	37 (41.6)	48 (35.0)
			
	***Diabetes***	14 (15.7)	25 (18.2)

* During the health record review, 8 patients were discovered to be ineligible and 1 audit was incomplete. Therefore the denominator for health record review data is 89 for the non-attenders and 136-7 for attenders

† p < 0.05

In the mail contact group, 8 letters were returned to sender. In the telephone contact group, all patients were reached in three tries or called back, and 4 were discovered to be ineligible on contact.

Overall, 58.3% (137/235) of invited patients attended a session. In the group invited by mail, 44.0% (51/116) patients attended, compared to 72.3% (86/119) among those invited by telephone. In univariate analyses, patients invited by telephone were significantly more likely to attend than were those invited by mail (OR 3.3; 95%CI 1.9–5.7; *p *< 0.001), and patients with a family history of cardiovascular disease noted in their chart were also significantly more likely to attend (OR 1.9; 95%CI 1.1–3.5; *p *= 0.049). In multivariate logistic regression modelling (n = 226), factors remaining significantly associated with attendance were invitation method (telephone) (OR 3.9, 95%CI 2.2–7.0; p < 0.001) and family history of cardiovascular disease recorded in the health record (OR 2.0, 95%CI 1.0–3.7; *p *= 0.38) (see Table 3).

**Table 3:** Univariate and multivariate analyses to detect an association between specific variables and attendance at a session.

**Variable**		**Attending (%)**	**Univariate analyses OR (95% CI)**	Multivariate analyses OR (95% CI)*
Invitation Method	***Mail***	51 (44.0)	1.0†	1.0†
	***Telephone***	86 (72.3)	3.3 (1.9–5.7) ‡	3.9 (2.2–7.0) ‡
				
Age (min 65 – max 96 yrs)	***65–74***	58 (58.0)	1.0†	
	***75+***	79 (58.5)	1.0 (0.6–1.7)	
				
Gender	***Female***	72 (59.6)	1.0†	
	***Male***	65 (59.0)	1.1 (0.6–1.8)	
				
Diagnosis of Hypertension§	***No***	76 (63.9)	1.0†	
	***Yes***	60 (56.6)	0.7 (0.4–1.3)	
				
Family physician-reported cardiovascular risk factors||	***Smoking***	87 (61.3)	1.0†	
		50 (59.5)	0.9 (0.5–1.6)	
				
	***Family history***	88 (56.1)	1.0†	1.0†
		49 (71.0)	1.9 (1.0–3.5) ‡	2.0 (1.0–3.7) ‡
				
	***High cholesterol***	89 (63.1)	1.0†	
		48 (56.5)	0.8 (0.4–1.3)	
				
	***Diabetes***	112 (59.9)	1.0†	
		25 (64.1)	1.2 (0.6–2.4)	

* model based on 226 cases with data from health record review

† reference category

‡ p < 0.05

§ based on 225 cases with data from health record review

|| based on 226 cases with data from health record review

Of the patients who attended a session and completed the questionnaire (n = 130), 80.0% (95/119) indicated an interest in attending again and 70.2% (85/121) preferred a session in the morning. The preference expressed for different invitation methods was close to evenly divided (49.6% mail and 50.4% telephone) and there was a tendency for patients to state a preference for the type of invitation they had actually received, mail (72.1%; 31/43) or telephone (64.3%; 45/70), with more patients contacted by telephone attending.

Mailing costs included postage ($0.47 × 116 patients = $54.52), stationary/printing ($8.50) and research assistant time to prepare the letters and correct addressing problems (4 hrs × $12/hr = $48.00). The telephone cost was for research assistant time to prepare a patient list and calling log (2 hrs × $12/hr = $24.00), and practice staff time (6 hrs at $25/hr = $150.00). Since 51 patients invited by mail attended, the cost per successful recruitment of one patient was $111.02 / 51 = $2.18. Of patients invited by telephone, 86 attended, for a per-patient cost of $174.00 / 86 = $2.02, indicating a minimal difference in efficiency.

## Discussion

The pharmacy sessions were well attended, indicating that organized community-based blood pressure monitoring with feedback to the family physician is feasible, and could enhance the diagnosis and management of hypertension by increasing the number of accurate readings available to the physician, and raising awareness among older adults of cardiovascular risk factors. The pilot demonstrated the advantage of incorporating invitation by telephone rather than mail, however the attendance rates for both groups and preferences reported by attendees demonstrated the success and acceptance of either method. Although attendance was higher in the telephone group, results also support use of mailed invitations, which are more easily implemented on a larger scale. The cost analysis demonstrated a minimal difference, and patient preference among attenders reflected the acceptability of either invitation method.

These findings are in keeping with similar studies of reminder strategies for preventive services in family practice. One study found an equal procedure completion rate among patients 15 years of age or older (n = 5883) of five preventive procedures with use of letter and telephone reminders, which was higher than that achieved through computerized physician reminders [15]. Procedure completion rates in both the letter and telephone groups were 42.0%, and letters were found to be more cost-effective.

Attendance at the Dundas sessions was higher, at 58.3% of invited patients on average, than might be expected from a single contact, particularly since participation involved attending one of a limited number of sessions held on specific days at five community pharmacies. The excellent attendance in response to a single letter or telephone call from the physician's office demonstrates that engaging older adults to participate in community-based initiatives, in cooperation with family physicians, is an effective and low-cost approach to cardiovascular health promotion.

Only two factors (invitation method and family history of cardiovascular disease) entered into the logistic regression model accounted for a significant portion of the variance in attendance, however these results are reassuring in the context of this population-health program. It was expected that male patients or very elderly patients might be less likely to attend community-based blood pressure monitoring, however, logistic regression analysis reveals that the program achieved good coverage of all patients, across the key variables of age, gender, hypertensive status, and cardiovascular risk factors.

A number of limitations of this pilot could be remedied in a larger study. For instance, the results of this study in one family physician's practice may not be generalizable to other practices, the success of one nurse in inviting patients may not be representative, and the limited time period in which the sessions were offered may have precluded attendance by some patients.

## Conclusion

While the attendance in response to a telephone invitation was higher, response to a single letter was substantial. Contact by the family physicians office was effective in encouraging older patients to participate in community-based assessment of cardiovascular risk factors, with feedback of results to the family physician. The pilot provided important perspectives toward expanding the program to a larger number of family physician practices, pharmacies, and older adults.

## Competing interests

The author(s) declare that they have no competing interests.

## Authors' contributions

All authors reviewed the manuscript and contributed to its critical revision for important intellectual content. TK contributed to the study design and was responsible for the analysis and interpretation of data and preparation of the manuscript. JK contributed to the study design and supervised the analyses. LC contributed to the study design. CL was a co-investigator contributing to the study design. LD contributed to the study design and recruited pharmacists. BM contributed to the study design and was responsible for training volunteers, coordinating the sessions, and data collection. CP is a co-investigator contributing to the study design. JEW contributed to the study design and invited patients from his practice.

## Pre-publication history

The pre-publication history for this paper can be accessed here:


